# Approaches to screening for hyperglycaemia in pregnant women during and after the COVID‐19 pandemic

**DOI:** 10.1111/dme.14380

**Published:** 2020-09-21

**Authors:** C. L. Meek, R. S. Lindsay, E. M. Scott, C. E. Aiken, J. Myers, R. M. Reynolds, D. Simmons, J. M. Yamamoto, D. R. McCance, H. R. Murphy

**Affiliations:** ^1^ Wellcome Trust‐MRC Institute of Metabolic Science Metabolic Research Laboratories University of Cambridge Cambridge UK; ^2^ Diabetes in Pregnancy Team Cambridge University Hospitals Cambridge UK; ^3^ Department of Clinical Biochemistry Cambridge University Hospitals Addenbrookes’s Hospital Cambridge UK; ^4^ Institute of Cardiovascular and Medical Sciences British Heart Foundation Glasgow Cardiovascular Research Centre University of Glasgow Glasgow UK; ^5^ Department of Population and Clinical Sciences Leeds Institute of Cardiovascular and Metabolic Medicine University of Leeds Leeds UK; ^6^ Maternal and Fetal Health Research Centre University of Manchester St Mary's Hospital Manchester UK; ^7^ Centre for Cardiovascular Science Queen's Medical Research Institute Edinburgh UK; ^8^ School of Medicine Western Sydney University Campbelltown NSW Australia; ^9^ Departments of Medicine and Obstetrics and Gynaecology University of Calgary Calgary Canada; ^10^ Regional Centre for Endocrinology and Diabetes Belfast UK; ^11^ Norwich Medical School Bob Champion Research and Education Building University of East Anglia Norwich UK; ^12^ Division of Women’s Health Kings College London London UK

## Abstract

**Aim:**

To evaluate the diagnostic and prognostic performance of alternative diagnostic strategies to oral glucose tolerance tests, including random plasma glucose, fasting plasma glucose and HbA_1c_, during the COVID‐19 pandemic.

**Methods:**

Retrospective service data (Cambridge, UK; 17 736 consecutive singleton pregnancies, 2004–2008; 826 consecutive gestational diabetes pregnancies, 2014–2019) and 361 women with ≥1 gestational diabetes risk factor (OPHELIA prospective observational study, UK) were included. Pregnancy outcomes included gestational diabetes (National Institute of Health and Clinical Excellence or International Association of Diabetes and Pregnancy Study Groups criteria), diabetes in pregnancy (WHO criteria), Caesarean section, large‐for‐gestational age infant, neonatal hypoglycaemia and neonatal intensive care unit admission. Receiver‐operating characteristic curves and unadjusted logistic regression were used to compare random plasma glucose, fasting plasma glucose and HbA_1c_ performance.

**Results:**

Gestational diabetes diagnosis was significantly associated with random plasma glucose at 12 weeks [area under the receiver‐operating characteristic curve for both criteria 0.81 (95% CI 0.79–0.83)], fasting plasma glucose [National Institute of Health and Clinical Excellence: area under the receiver‐operating characteristic curve 0.75 (95% CI 0.65–0.85); International Association of Diabetes and Pregnancy Study Groups: area under the receiver‐operating characteristic curve 0.92 (95% CI 0.85–0.98)] and HbA_1c_ at 28 weeks' gestation [National Institute of Health and Clinical Excellence: 0.83 (95% CI 0.75–0.90); International Association of Diabetes and Pregnancy Study Groups: 0.84 (95% CI 0.77–0.91)]. Each measure predicts some, but not all, pregnancy outcomes studied. At 12 weeks, ~5% of women would be identified using random plasma glucose ≥8.5 mmol/l (sensitivity 42%; specificity 96%) and at 28 weeks using HbA_1c_ ≥39 mmol/mol (sensitivity 26%; specificity 96%) or fasting plasma glucose ≥5.2–5.4 mmol/l (sensitivity 18–41%; specificity 97–98%).

**Conclusions:**

Random plasma glucose at 12 weeks, and fasting plasma glucose or HbA_1c_ at 28 weeks identify women with hyperglycaemia at risk of suboptimal pregnancy outcomes. These opportunistic laboratory tests perform adequately for risk stratification when oral glucose tolerance testing is not available.

**What's new?**

- Screening procedures for hyperglycaemia in pregnancy have temporarily changed during the COVID‐19 pandemic as oral glucose tolerance tests are challenging to conduct with social distancing measures in place, public transport restrictions and reduced clinical capacity.
- National recommendations for Australia, Canada, New Zealand and the UK propose alternative opportunistic screening strategies using HbA_1c_, random plasma glucose and fasting plasma glucose (FPG), performed with routine antenatal bloods at booking and 28 weeks.
- We identified that random plasma glucose, HbA_1c_ and FPG were all associated with gestational diabetes diagnosis and that all three tests can predict obstetric and neonatal outcomes but lack the evidence base and sensitivity of an oral glucose tolerance test.
- Future work should prioritize inclusive screening strategies which identify women at highest risk of materno‐fetal complications and systematic procedures for longer‐term cardio‐metabolic follow‐up.

## 1 INTRODUCTION

Gestational diabetes mellitus (GDM) affects approximately 5% of pregnant women in the UK and is associated with perinatal morbidity, including large‐for‐gestational‐age (LGA) infants, complicated deliveries and neonatal hypoglycaemia.^1^ The oral glucose tolerance test (OGTT) is currently the recommended approach to the diagnosis of GDM in the UK and internationally.^2^, ^3^ Early in 2020, a novel virus, SARS‐CoV‐2, reached pandemic levels of worldwide infection.^4^ In the UK, pregnant women have been advised to remain in self‐isolation for at least 12 weeks except for essential excursions for food, healthcare and health reasons (including outdoor daily exercise), with similar restrictions internationally. This, alongside public transport limitations, especially during peak hours, social distancing and laboratory requirements, has made it challenging for healthcare providers to implement routine OGTTs. Furthermore, with staff shortages due to self‐isolation, illness or redeployment, the clinical capacity for managing large numbers of pregnant women with milder forms of hyperglycaemia has been reduced. An alternative approach to screening for severe forms of maternal hyperglycaemia during the COVID‐19 pandemic was required for urgent implementation.^5^ The intention was not aimed at identifying an equivalent group of women as those detected by OGTT, but rather to identify pregnant women with the highest glucose levels for whom specialist management remained essential throughout the pandemic. The alternative strategy recommends glucose testing during other hospital or community appointments to minimize additional clinical contacts.^5^

The OGTT has well recognized limitations in terms of test reproducibility, tolerability and seasonal influences,^6^, ^7^ but remains the most commonly recommended diagnostic strategy for GDM.^2^, ^3^ Excess fetal growth acceleration is detectable from 20 weeks’ gestation, predating diagnosis of GDM.^8^ However, approximately half of women with identified risk factors for GDM, do not have an OGTT performed in routine care settings. Importantly, among women with stillbirths, an OGTT was only performed in 38% of women with GDM risk factors.^9^ A nationwide UK audit confirmed that OGTT testing was least likely to be performed in obese women and those from higher‐risk ethnic groups.^10^ Although some women may refuse OGTT testing, it is clear that screening for GDM is not consistently implemented in accordance with guideline recommendations.^6^, ^10^

A recent case–control study identified a 44% greater risk of stillbirth in women with risk factors when an OGTT was not performed.^9^ Likewise, women with raised fasting plasma glucose (FPG) levels who were not diagnosed with GDM experienced a fourfold greater risk of stillbirth.^9^ Women who were appropriately screened and treated had no increased risk of stillbirth.^9^ This failure to effectively implement OGTT in routine clinical care settings even prior to the pandemic leaves women exposed to potentially modifiable risks for stillbirth.

Relatively little research has focused on identifying a suitable alternative to the OGTT. Some novel technologies and biomarkers show promise in small studies, but do not have proven diagnostic performance or wide availability. Performing an OGTT at home or replacing it with glucometer or continuous glucose monitoring readings was not feasible for widespread implementation during the pandemic.

The interim testing strategy for hyperglycaemia in pregnancy aimed to: (1) identify women with the most severe hyperglycaemia for prioritization of resources towards those at highest risk of suboptimal obstetric and neonatal outcomes; (2) fit around women’s routine antenatal visits at 12, 20 and 28 weeks (for blood tests or ultrasound scans), avoiding additional visits to healthcare environments; (3) use established laboratory methods with good analytical performance and wide availability; (4) avoid increasing clinical workload during staff shortage (prioritizing test specificity over sensitivity); (5) be simple to understand (by non‐specialists) and easy to rapidly implement across healthcare environments; and (6) use an existing evidence base to support it and to enable audit after the pandemic.

Several diagnostic strategies for hyperglycaemia in pregnancy have been suggested for use during the COVID‐19 pandemic (Table 1). The UK approach advises measuring HbA_1c_ and random plasma glucose in all women with risk factors for GDM (previous GDM, BMI >30 kg/m^2^, high‐risk ethnic groups, family history of diabetes, previous macrosomic baby >4.5 kg) at the first antenatal visit (Table 4). Women with HbA_1c_ levels ≥48 mmol/mol or random plasma glucose levels ≥11.1 mmol/l are managed as having pre‐gestational, most commonly type 2 diabetes. Women with HbA_1c_ levels 41–47 mmol/mol or random plasma glucose levels 9–11.0 mmol/l are managed as having early‐onset GDM. Women with HbA_1c_ <41 mmol/mol and random plasma glucose <9 mmol/l are retested at 28 weeks, with a repeat HbA_1c_ and FPG (if possible) or random plasma glucose performed. Women with FPG ≥5.6 mmol/l, HbA_1c_ ≥39 mmol/mol or random plasma glucose ≥9 mmol/l are managed as having GDM. The pandemic testing procedures do not exclude GDM and further testing should be performed, at any gestational age, in women with glycosuria, symptoms of diabetes, or ultrasound features of LGA infant or polyhydramnios.^5^ Similar strategies were recommended in Australia, Canada and New Zealand (Table 1).^11^, ^12^, ^13^

**Table 1:** Recommendations from Australia, Canada, New Zealand and the UK for identification of hyperglycaemia in pregnancy during the COVID‐19 pandemic [5,11–13]

	Australia	Canada	New Zealand	UK
Glucose measures at 12 weeks	HbA_1c_	HbA_1c_ or FPG	HbA_1c_	HbA_1c_ and random plasma glucose
Interpretation of early pregnancy glucose measures	HbA_1c_ >41 mmol/mol diagnosed with GDM	HbA_1c_ before 20 weeks to identify overt diabetes in high‐risk women only	HbA_1c_ >40 mmol/mol: refer to specialist clinic	HbA_1c_ 41–47 mmol/mol or random plasma glucose 9–11 mmol/l: manage as early GDM. HbA_1c_ ≥ 48 mmol/mol or random plasma glucose >11.1 mmol/l: manage as likely type 2 diabetes.
Glucose measures at 28 weeks	OGTT or FPG	Standard two‐step protocol or HbA_1c_ and random plasma glucose	Standard 2‐h OGTT or FPG if HbA_1c_ at 12 weeks <41 mmol/mol	HbA_1c_ and random plasma glucose or FPG (FPG if possible according to clinical capacity)
Interpretation of glucose measures at 28 weeks	Standard OGTT (fasting ≥ 5.1 mmol/l; 1‐h ≥ 10 mmol/l or 2‐h ≥ 8.5 mmol/l ) or FPG alone ≥ 5.1 mmol/l	HbA_1c_ ≥5.7% (39 mmol/mol) or random plasma glucose ≥11.1 mmol/l: diagnosed with GDM	FPG ≥5.0 mmol/l: treat as GDM. FPG 4.5–5.0 mmol/l: SMBG for 2 weeks and dietetic support, especially if risk factors for GDM	HbA_1c_ ≥ 39 mmol/mol or random plasma glucose >9 mmol/l or FPG ≥ 5.6 mmol/l: diagnosed with GDM.
Women with previous GDM	Can have standard testing schedule or be assumed to have GDM and started on self‐monitoring at home	Not specifically mentioned. Standard testing pathway applies	Start SMBG from 12 weeks	Treat as GDM from 12 weeks if HbA_1c_ 41–47 mmol/mol or random plasma glucose 9–11 mmol/l. If HbA_1c_ <41 mmol/mol and random plasma glucose <9 mmol/l: standard testing at 28 weeks applies
Provisions for testing at other times	Clinicians to use clinical judgement about suitability of testing	Testing can be repeated in later pregnancy is there is a high clinical suspicion of diabetes	Not mentioned	Test in the presence of heavy glycosuria (2+ or above), diabetes symptoms or according to scan features (LGA fetus or polyhydramnios)
Postpartum testing	With OGTT to be delayed by 6–12 months. For women at high risk of type 2 diabetes, consider self‐monitoring at home or HbA_1c_ 4–6 months	Defer until after the pandemic is over	Not mentioned	HbA_1c_ at 3–6 months after birth

FPG, fasting plasma glucose; GDM, gestional diabetes; LGA, large‐for‐gestational‐age; OGTT, oral glucose tolerance test; SMBG, self‐monitoring of blood glucose.

The aim of the present study was to provide evidence‐based recommendations for a pragmatic diagnostic strategy for hyperglycaemia in pregnancy, applicable during the COVID‐19 pandemic.

## 2 METHODS

Data from complete and ongoing studies (Table 2; methodology and patient characteristics) were used to assess diagnostic performance of potential glucose measures including HbA_1c_, random plasma glucose, FPG and 1‐h and 2‐h plasma glucose after a 75‐g OGTT performed in a real‐world clinical setting.

**Table 2:** Characteristics of women included in each dataset

Study characteristics	Older CUHFT cohort	Recent CUHFT cohort	OPHELIA cohort
	*n* = 17736	*n* = 826	*n* = 361
Time period covered	2004–2008	2014–2019	Oct 2019–ongoing
Study design	Retrospective service evaluation	Retrospective service evaluation	Prospective observational study
Population	All singleton pregnancies with liveborn infants	All singleton pregnancies with GDM diagnosed according to IADPSG criteria	Singleton pregnancies with ≥1 positive NICE risk factor
Centres	Single centre	Single centre	Four East of England centres
Who had a random plasma glucose at 12 weeks?	All women offered, results available in 72%^†^	Not applicable	Not applicable
Who had a 75‐g OGTT at 24–28 weeks?	Women with a 24‐week 50‐g glucose challenge result >7.7 mmol/l	Women with ≥1 GDM risk factor	Women with ≥1 GDM risk factor
GDM diagnostic criteria	1998 WHO [31]	IADPSG [2]	NICE [3]
Treatment offered, *n*/*N* (%)	776/17736 (4.4)	826/826 (100)	30/331 (8.3)
**Maternal characteristics**			
Mean (sd) maternal age years	30.9 (5.6)	33.6 (5.4)	31.7 (4.9)
Mean (sd) pre‐pregnancy BMI, kg/m^2^	24.8 (5.0)	29.4 (7.5)	33.0 (6.7)
Primiparous, *n* (%)	9895 (56)	283 (34)	132 (37)
Ethnicity, *n* (%)		*n*=670	*n*=345
White	15934 (90)	531 (79)	315 (91)
Black	258 (1.5)	14 (2.1)	29 (8.4)
Asian	899 (5.1)	111 (17)	1 (0.3)
Other	644 (3.6)	14 (2.1)	0 (0.0)
Maternal smoking, *n* (%)	1643 (9.3)	61/747 (8.2)	N/A
Mean (sd) random plasma glucose mmol/l at 12 weeks, mmol/l	5.8 (1.4)	N/A	N/A
Mean (sd) HbA_1c_ at 28 weeks, mmol/mol	N/A	35.9 (4.9)	32.5 (3.6)
**OGTT at 28 weeks**	*n* = 3848	*n* = 821–824	*n* = 359–360
Mean (sd) Fasting glucose (OGTT time 0)	4.5 (0.6)	4.9 (0.7)	4.4 (0.5)
Mean (sd) OGTT time 60 mins	8.5 (1.9)	10.6 (1.5)	N/A
Mean (sd) OGTT time 120 mins	6.9 (1.6)	7.6 (1.4)	5.8 (1.4)
**Pregnancy outcomes**	*n* = 17001–17736	*n* = 817‐826	*n* = 278
Mean (sd) estimated gestational age at birth, weeks	39.2 (2.0)	38.5 (1.4)	39.4 (1.5)
LGA infant, *n* (%)	2112 (1)	134 (16)	N/A
Caesarean section, *n* (%)	5005 (28)	342 (41)	N/A
Neonatal hypoglycaemia, *n* (%)	N/A	386 (47)	N/A
NICU admission, *n* (%)	1071 (6.1)	124 (15)	N/A

CUHFT, Cambridge University Hospital NHS Foundation Trust; FPG, fasting plasma glucose; LGA, large‐for‐gestational‐age; NICU, neonatal intensive care unit.

Neonatal hypoglycaemia defined as neonatal glucose <2.6 mmol/l at least 4 h after birth. NICU admission defined as >24 h.

^†^ No difference between women with and without a random plasma glucose.^31^

Gestational diabetes diagnosis was classified according to the criteria of the UK National Institute for Health and Care Excellence (NICE; 0 min ≥5.6 mmol/l; 120 min ≥7.8 mmol/l)^3^ and the International Association of Diabetes and Pregnancy Study Groups (IADPSG), adopted by the WHO (IADPSG/WHO; 0 min ≥5.1; 60 min ≥10.0; 120 min ≥8.5 mmol/l)^2^.

Older (2004–2008) and more recent data (2014–2019) from approved service evaluations at Cambridge University Hospital NHS Foundation Trust (CUHFT) used for this study have been previously described.^14^, ^15^ In brief, the older cohort includes 17 736 consecutive women with singleton pregnancies, with random plasma glucose performed at booking followed by a universal 50‐g glucose challenge test at 24 weeks. Women with a 50‐g glucose challenge result of >7.7 mmol/l had a 75‐g OGTT at 28 weeks’ gestation (*n* = 3848) and were offered treatment (776/17 736; 4.4%) in line with NICE guidance.^16^ A minority (<5%) had an OGTT using capillary rather than venous blood. The more recent cohort included 826 consecutive women with GDM (risk factor screening; 75‐g OGTT 24–28 weeks using IADPSG/WHO criteria, 20 October 2014 to 31 January 2019), who received standard clinical management. Detailed information on pregnancy outcomes was gathered from electronic medical records as part of an ongoing service evaluation.^14^

In addition, data were included from 361 women with one or more risk factors for GDM,^3^ recruited from an ongoing multicentre prospective study, OPHELIA (Observational study of Pregnancy Hyperglycaemia, Endocrine causes, Lipids, Insulin and Autoimmunity; REC 18/LO/0477; researchregistry no.5528). Briefly, women with a singleton pregnancy and one or more GDM risk factors^3^ were invited for a 24–28‐week 75‐g OGTT with measurement of HbA_1c_. A total of 8.3% of women had GDM (NICE criteria) and were offered treatment. Pregnancy outcome data from this ongoing study are not available.

Assessment of neonatal outcomes was performed using the older and recent CUHFT datasets. We chose outcomes which are directly related to hyperglycaemia, are consistently measured during GDM pregnancies, are potentially modifiable by standard clinical management,^1^, ^17^ and which have a defined impact on healthcare costs.^3^

Large for gestational age was defined as having a birth weight >90^th^ centile using locally derived standardized centiles adjusted for infant sex and gestational age.^7^ Neonatal hypoglycaemia was defined as a capillary blood glucose level <2.6 mmol/l on more than one occasion at least 4 h after birth. Admission to the neonatal intensive care unit (NICU) was defined as admission for 24 h or longer.

The ability of each glucose‐related variable to predict GDM diagnosis was assessed using receiver‐operating characteristic (ROC) curves. Unadjusted logistic regression identified associations between glucose measures and pregnancy outcomes (odds ratios and 95% CIs reported). Missing data were not imputed. A statistical significance level of 5% was used throughout.

### 2.1 Ethics

The OPHELIA study was approved by the London and Westminster research ethics committee (REC 18/LO/0477; research registry no.5528). The CUHFT data were collected as part of approved service evaluations. Further ethical approval was not required for this analysis.

## 3 RESULTS

### 3.1 Prediction of gestational diabetes diagnosis and association with pregnancy outcomes

**FIGURE 1:**
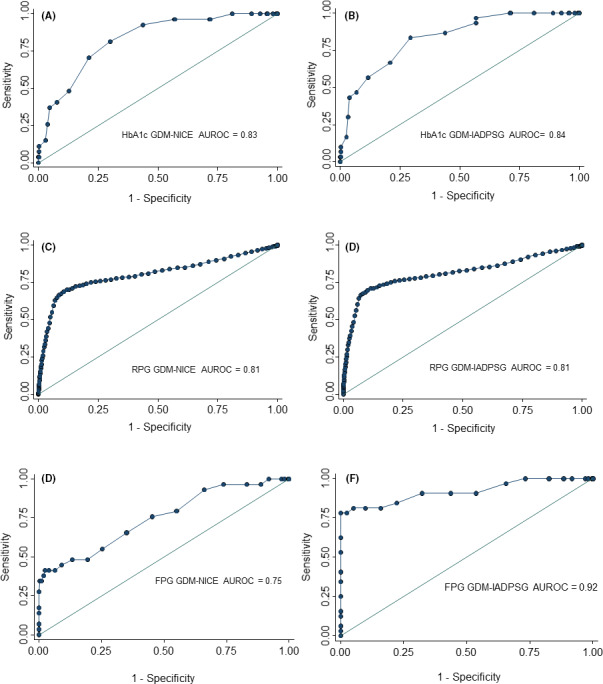
Receiver‐operating characteristic curves for (A & B) HbA_1c_ at 28 weeks, (C & D) random plasma glucose at booking and fasting plasma glucose (FPG) at 28 weeks for predicting gestational diabetes diagnosis according to NICE and IADPSG criteria.

All glucose measures were significantly associated with GDM diagnosis on ROC curves (Table 3 and Fig. 1). Among the alternative glucose measures, HbA_1c_ at 28 weeks and random plasma glucose at booking (12±2 weeks) both performed reasonably well, with areas under the ROC (AUROCs) of 0.83 (95% CI 0.75–0.90, OPHELIA) and 0.81 (95% CI 0.79–0.83, older CUHFT cohort) to predict NICE‐defined GDM.^4^ The AUROC for random plasma glucose at booking was comparable for GDM as defined ether by NICE or IADPSG criteria. FPG was slightly less predictive for GDM defined by the NICE criteria [AUROC 0.75 (95% CI 0.65–0.85), OPHELIA], but strongly predicted GDM defined according to the IADPSG criteria [AUROC 0.92 (95% CI 0.85–0.98) OPHELIA]. All glucose measures studied had associations with one or more outcomes (Table 3).

**Table 3:** Associations between glucose measures, diagnosis of gestational diabetes and pregnancy outcomes

Study	Population	Total, *n*	OGTT, *n*	Design	Outcome	Random plasma glucose 12 weeks	HbA_1c_ 28 weeks	FPG 28 weeks	OGTT time 60 28 weeks	OGTT time 120 28 weeks
Diagnosis: receiver‐operating characteristic curves						AUROC (95% CI)	AUROC (95% CI)	AUROC (95%CI)	AUROC (95%CI)	AUROC (95%CI)
OPHELIA	Antenatal population with ≥1 GDM risk factor	361	361	Prospective	NICE‐GDM	No data	0.83 (0.75‐‐0.90)***	0.75 (0.65‐0.85)***	no data	0.93 (0.86‐1.00)***
		361	361	Prospective	IADPSG‐GDM	No data	0.84 (0.77‐‐0.91)***	0.92 (0.85‐0.98)***	no data	0.83 (0.75‐0.92)***
Older CUHFT	Antenatal population with positive glucose challenge test^†^	17736	3764	Retrospective	NICE‐GDM	0.81 (0.79‐‐0.83)***	No data	0.69 (0.67‐0.71)***	0.83 (0.82‐0.85)***	0.99 (0.98‐0.99)***
		17736	3764	Retrospective	IADPSG‐GDM	0.81 (0.79‐‐0.83)***	No data	0.79 (0.77‐0.80)***	0.93 (0.92‐0.94)***	0.85 (0.84‐0.86)***

Diagnosis: unadjusted logistic regression						OR (95%CI)	OR (95%CI)	OR (95%CI)	OR (95%CI)	OR (95%CI)
Older CUHFT	Antenatal population with positive glucose challenge at 24 weeks	17736	3764	Retrospective	NICE‐GDM	2.35 (2.24‐‐2.46)***	No data	4.84 (4.13‐5.67)***	2.29 (2.16‐2.44)***	348.82 (200.76‐606.07)***
		17736	3764	Retrospective	IADPSG‐GDM	2.45 (2.34‐‐2.57)***	No data	16.00 (12.98‐19.71)***	5.76 (5.11‐6.49)***	3.66 (3.35‐4.01)***
Recent CUHFT	Women with ≥1 GDM risk factor according to the IADPSG criteria	826	826	Prospective	NICE‐GDM	No data	*n*/a	*n*/a	no data	*n*/a
		826	826	Prospective	IADPSG‐GDM	No data	*n*/a	*n*/a	no data	*n*/a

Pregnancy outcomes: unadjusted logistic regression						OR (95% CI)	OR (95% CI)	OR (95% CI)	OR (95% CI)	OR (95% CI)
Older CUHFT	Antenatal population with positive glucose challenge at 24 weeks	17736	3764	Retrospective	LGA infant	1.10 (1.06‐‐1.13)***	No data	1.87 (1.63‐2.14)***	1.11 (1.06‐1.15)***	1.04 (0.99‐1.10)
		17736	3764	Retrospective	CS	1.15 (1.21‐1.17)***	No data	1.48 (1.32‐1.67)***	1.10 (1.06‐1.14)***	1.11 (1.06‐1.15)***
		17736	3764	Retrospective	NH	No data	No data	No data	No data	No data
		17736	3764	Retrospective	NICU	1.06 (1.02‐1.11)**	No data	1.03 (0.82‐1.28)	1.02 (0.95‐1.10)	1.05 (0.97‐‐1.14)
Recent CUHFT	Women with ≥1 GDM risk factor according to the IADPSG criteria	826	826	Prospective	LGA infant	no data	1.04 (1.00‐‐1.08)*	1.28 (1.01‐‐1.62)*	No data	1.02 (0.92‐‐1.14)
		826	826	Prospective	CS	no data	1.04 (1.01‐‐1.08)**	1.06 (0.88‐‐1.28)	No data	1.04 (0.95‐‐1.13)
		826	826	Prospective	NH	no data	1.05 (1.02‐‐1.08)**	0.95 (0.79‐‐1.15)	No data	1.00 (0.92‐‐1.08)
		826	826	Prospective	NICU	no data	1.02 (0.98‐‐1.06)	1.21 (0.94‐‐1.54)	No data	0.93 (0.83‐‐1.04)

AUROC, area under the receiver‐operating characteristic curve; CS, Caesarean section; CUHFT, Cambridge University Hospital NHS Foundation Trust; FPG, fasting plasma glucose; GDM, gestational diabetes; IADPSG, International Association of Diabetes and Pregnancy Study Groups; LGA, large‐for‐gestational‐age; NICE, National Institute for Health and Care Excellence; NICU, neonatal intensive care unit; NH, neonatal hypoglycaemia; NICU. neonatal intensive care unit; OR, odds ratio.

NH defined as neonatal glucose <2.6 mmol/l at least 4 h after birth. NICU admission defined as >24 h. OPHELIA included 361 women (8.3% with NICE‐defined GDM) all offered standard clinical management. Older CUHFT data included 4.3% of women (4.5% using current NICE GDM criteria) offered standard clinical management. The recent CUHFT data includes women with IADPSG‐defined GDM, all of whom were offered standard clinical management. ORs were calculated using continuous measures. *P < 0.05; **P<0.01; ***P<0.001. ^†^Glucose >7.8 mmol/l, 1 h after a universal 50‐g glucose challenge at 24 weeks' gestation.

### 3.2 Assessment of potential thresholds

The sensitivity and specificity of thresholds for random plasma glucose, HbA_1c_ and FPG to predict GDM using NICE or IADPSG diagnostic criteria are given in Table 4. For example, to identify a similar proportion of women as detected by the NICE criteria (~5%) would require a 12‐week random plasma glucose of ≥8.5 mmol/l (42% sensitivity, 96% specificity; older CUHFT cohort), a 28‐week HbA_1c_ of ≥39 mmol/mol (26% sensitivity, 96% specificity; OPHELIA); or an FPG of ≥5.2–5.4 mmol/l (sensitivity 18–41%, specificity 97–98%; OPHELIA or older CUHFT).

**Table 4:** Sensitivity and specificity of various thresholds for prediction of National Institute of Health and Care Excellence‐ and International Association of Diabetes and Pregnancy Study Groups‐defined gestational diabetes [3,4]

HbA_1c_ at 28 weeks vs OGTT at 28 weeks: OPHELIA data, *n*=340								
Threshold	NICE‐GDM AUC 0.83 (95% CI 0.75, 0.90)				IADPSG‐GDM AUC 0.84 (95% CI 0.77, 0.91)			
	Sensitivity, %	Specificity, %	*n*	% positive	Sensitivity, %	Specificity, %	*n*	% positive
> 32 mmol/mol (5.1%)	96	43	204	56	93	43	204	60
> 33 mmol/mol (5.2%)	93	56	162	45	87	56	162	48
> 34 mmol/mol (5.3%)	82	70	116	32	83	71	116	34
> 35 mmol/mol (5.4%)	70	79	85	24	67	79	85	25
> 36 mmol/mol (5.4%)	48	87	53	15	57	88	53	16
> 37 mmol/mol (5.5%)	41	92	35	9.7	47	93	35	10
> 38 mmol/mol (5.6%)	37	95	25	6.9	43	96	25	7.3
> 39 mmol/mol (5.7%)	26	96	19	5.3	30	97	19	5.6
> 40 mmol/mol (5.8%)	15	97	13	3.6	17	97	13	3.8

Random plasma glucose at 12 weeks vs OGTT at 28 weeks: older CUHFT data, *n*=17 736								
Threshold	NICE‐GDM AUC 0.81 (95% CI 0.79, 0.83)				IADPSG‐GDM AUC 0.81 (95% CI 0.79, 0.83)			
	Sensitivity	Specificity	*n*	% positive	Sensitivity	Specificity	*n*	% positive
> 7.0 mmol/l	73	83	3487	20	74	83	3487	20
> 7.5 mmol/l	69	89	2340	13	70	90	2340	13
> 7.8 mmol/l	65	92	1835	10	67	93	1835	10
> 8.0 mmol/l	60	94	1541	8.7	60	94	1541	8.7
> 8.5 mmol/l	42	96	947	5.3	43	97	947	5.3
> 9.0 mmol/l	29	98	576	3.2	30	98	576	3.2
> 9.5 mmol/l	19	99	340	1.9	20	99	340	1.9
> 10.0 mmol/l	11	100	171	1.0	12	100	171	1.0

Fasting plasma glucose at 28 weeks vs OGTT at 28 weeks: OPHELIA data, *n* = 360								
Cutpoint	NICE‐GDM AUC 0.75 (95% CI 0.65, 0.85)				IADPSG‐GDM AUC 0.92 (95% CI 0.85, 0.98)			
	Sensitivity	Specificity	*n*	% positive	Sensitivity	Specificity	*n*	% positive
> 4.5 mmol/l	65	65	135	38	91	68	135	38
> 5.0 mmol/l	41	94	33	9.2	78	98	33	9.2
> 5.1 mmol/l	41	96	25	6.9	78	100	25	6.9
> 5.2 mmol/l	41	98	20	5.6	63	100	20	5.6
> 5.3 mmol/l	38	98	17	4.7	53	100	17	4.7
> 5.4 mmol/l	35	99	13	3.6	41	100	13	3.6
> 5.5 mmol/l	35	100	11	3.1	34	100	11	3.1
> 5.6 mmol/l	28	100	8	2.2	25	100	8	2.2

Fasting plasma glucose at 28 weeks vs OGTT at 28 weeks: older CUHFT data, *n* = 3832								
Threshold	NICE‐GDM AUC 0.69 (95% CI 0.82, 0.85)				IADPSG‐GDM AUC 0.79 (95% CI 0.77, 0.80)			
	Sensitivity, %	Specificity, %	*n*	% positive	Sensitivity, %	Specificity, %	*n*	% positive
> 4.5 mmol/l	65	61	1758	46	73	66	1758	46
> 5.0 mmol/l	33	91	597	16	44	97	597	16
> 5.1 mmol/l	29	94	473	12	40	100	473	12
> 5.2 mmol/l	26	96	370	9.7	31	100	370	9.7
> 5.3 mmol/l	23	98	299	7.8	25	100	299	7.8
> 5.4 mmol/l	18	99	229	6.0	19	100	229	6.0
> 5.5 mmol/l	16	100	182	4.7	15	100	182	4.7
> 5.6 mmol/l	14	100	148	3.9	13	100	148	3.9

AUC, area under the curve; CUHFT, Cambridge University Hospital NHS Foundation Trust; GDM, gestational diabetes; IADPSG, International Association of Diabetes and Pregnancy Study Groups; NICE, National Institute of Health and Care Excellence; IADPSG, International Association of Diabetes and Pregnancy Study Groups; OGTT, oral glucose tolerance test.

Note that not all patients included underwent an OGTT.

### 3.3 Predictors for diabetes in pregnancy

Fasting plasma glucose at 28 weeks was the best predictor of diabetes in pregnancy [AUROC 0.9 (95% CI 0.86–0.95); older CUHFT), with a specificity of >90% and sensitivity of 50–70% at thresholds of ≥5.2 mmol/l (Table S1). Unfortunately, the number of women with HbA_1c_ >48 mmol/mol was not available and the number with IADPSG‐defined ‘overt diabetes’ was too small (*n* = 19) to draw any conclusions.

## 4 DISCUSSION

There are limited data available to support a change in diagnostic criteria for GDM in a real‐world clinical setting. Despite this, our results provide some evidence regarding the use of routine antenatal blood tests, random plasma glucose at 12 weeks' gestation and HbA_1c_ or FPG at 28 weeks' gestation for diagnosing hyperglycaemia in pregnancy during the COVID‐19 pandemic.^5^ These established, affordable, widely available laboratory tests have reasonable ability to predict diagnosis of GDM and to identify women at highest risk of suboptimal glycaemic, obstetric and neonatal outcomes. Although the sensitivity of these measures is substantially lower than that of the OGTT, the specificity is sufficient to allow targeted assessment of women at highest risk.

Despite differences between the universal and selective risk factor screening procedures and patient characteristics, the results show consistent associations between routine antenatal glucose measures, GDM diagnosis and clinically relevant obstetric and neonatal outcomes (Caesarean delivery, LGA infant, neonatal hypoglycaemia, neonatal intensive care unit admission). However, the women included in the present study were not blinded to their diagnosis, had GDM diagnosed using different screening pathways and diagnostic criteria, and had no measures of glycaemic control later in pregnancy. The treatment of patients within these clinical datasets will not affect diagnostic predictions but may have reduced associations with maternal or neonatal outcomes. The use of unadjusted regression reflects clinical decision‐making; adjusted odds ratios may show different associations. In addition, these datasets do not have sufficient ethnic diversity to fully represent national or international populations, with more women belonging to higher‐risk ethnic groups. The older CUHFT dataset is large but not all women underwent an OGTT to exclude GDM and none of the datasets used universal screening for GDM with an OGTT at 28 weeks. These results reflect test performance in high‐risk cohorts. The OPHELIA study reflects a high‐risk cohort, chosen according to risk factors^3^ (UK approach), while the older CUH cohort reflects a high‐risk cohort chosen from a two‐step strategy (used widely in USA).^5^ Although these results are not directly comparable to assessments of test performance in an unselected population, they are comparable to current clinical practice internationally. Although preventing stillbirth is a priority during the pandemic, as this outcome is so rare, the datasets provide insufficient data to allow this outcome to be assessed accurately.

Compared to an OGTT, random plasma glucose, HbA_1c_ and FPG assessments have less evidence to support their use in the diagnosis of GDM. The ability of a first‐trimester OGTT to predict pregnancy outcomes is also unclear. However, random plasma glucose performs surprisingly well as a first‐trimester predictive tool for later GDM diagnosis.^15^ An early‐pregnancy random plasma glucose also has consistent associations with Caesarean section, LGA infant, and neonatal intensive care unit admission, but the small odds ratios suggest that random plasma glucose alone lacks precision as a prognostic tool. The performance of and optimal thresholds for random plasma glucose in late pregnancy are unknown. Random plasma glucose was included pragmatically, alongside routine bloods, to minimize the logistical challenges associated with obtaining multiple early‐morning fasted samples within a short timeframe.

Fasting plasma glucose assessment, when performed as part of an OGTT at 24–28 weeks' gestation, has strong associations with the pregnancy outcomes attributed to fetal hyperinsulinism including primary Caesarean delivery, LGA infant and neonatal hypoglycaemia.^18^ The necessity of having a test in the fasting state, and therefore in the morning, is associated with non‐attendance.^6^ This was pertinent during the pandemic peak, when having all women attend during a small, timeframe was logistically difficult, and challenging for women without private transport. More data are required to better understand whether the requirement for an overnight fast and early‐morning attendance influences uptake of the OGTT, especially among socially disadvantaged women in real‐world clinical settings^6^

Unfortunately, although included in the UK, Canadian, Australian and New Zealand recommendations^5^, ^11^, ^12^, ^13^ to detect overt and/or pre‐gestational diabetes, we did not have data for HbA_1c_ in early pregnancy. Hughes *et al*.^19^ previously demonstrated that an early‐pregnancy HbA_1c_ ≥ 41 mmol/mol (5.9%) was predictive for diabetes and for identifying mothers and offspring at risk of complications.^19^ In the present study, we found that HbA_1c_ ≥39 mmol/mol (5.7%) at 28 weeks' gestation had good specificity (96%) and identified approximately 5% of women who were screened, a comparable number to those identified using the NICE or IADPSG criteria.^3^, ^4^ Outside pregnancy, HbA_1c_ is widely used both for diabetes diagnosis and glycaemic monitoring, and is highly predictive of diabetes complications.^20^, ^21^ However, accuracy is reliant on stable red cell turnover and the absence of haematological disease, iron deficiency or inherited haemoglobin variants.^21^, ^22^ In early pregnancy, red cell turnover increases, contributing to the well‐recognized non‐glycaemic reduction in HbA_1c_ in the late first/early second trimesters.^21^ HbA_1c_ is therefore a poor marker for individual glycaemia, but remains important for predicting obstetric and neonatal outcomes including preterm delivery, LGA infant and neonatal intensive care unit admission.^23^ The proposed screening strategy includes an early HbA_1c_ measurement to exclude overt diabetes and a 28‐week measurement for pragmatic reasons, with interpretation in conjunction with another glucose measure, either FPG or random plasma glucose.

Although the OGTT is the most widely used test for GDM diagnosis, this recommendation is largely based on research data with optimal pre‐analytical processing and analytical performance within specialist laboratory settings.^30^ The OGTT has poor reproducibility in real‐world clinical settings.^24^ Non‐pregnant individuals having two OGTTs within 1 week receive the same diagnosis of diabetes, prediabetes, or normal glucose tolerance on 27–80% of occasions.^25^ FPG values can vary by 10–30% in adults with normal glucose tolerance.^25^ The intra‐individual variation in OGTT glucose is predominantly determined by biological variation in normoglycaemic adults, with 95% of the test–retest variability of <15% for FPG and <46% for 2‐h post OGTT glucose.^26^ The diagnostic performance of an OGTT is pertinent during pregnancy, where substantial variability was described by O’Sullivan *et al*.^27^ more than 50 years ago. More recently, seasonal differences in OGTT performance have been highlighted: higher ambient temperatures may increase GDM diagnosis by ~30% in the UK summer.^7^ Pre‐analytical processing is also critical: a recent Australian study reported that early centrifugation (<10 min) was associated with almost a doubling in GDM diagnoses.^24^

A Canadian study found that almost 50% of women with GDM diagnosed using an OGTT were normoglycaemic using capillary glucose monitoring in daily life, emphasizing concerns about sensitivity.^28^ Continuous glucose monitoring reflects fetal exposure to maternal glycaemia during the 24‐h day, providing substantially more detailed glucose measurements compared to an OGTT or capillary glucose monitoring. Preliminary data have identified the continuous glucose monitoring glucose profiles associated with LGA infants in women with GDM, although further research is needed to determine the feasibility and performance of continuous glucose monitoring for GDM diagnosis.^29^

Despite its limitations, the OGTT has a strong evidence base in the diagnosis of GDM.^18^ The Hyperglycaemia and Adverse Pregnancy Outcomes (HAPO) study demonstrated consistent linear associations between maternal glucose concentrations during an OGTT with pregnancy outcomes.^18^ Furthermore, the OGTT identifies women who gain demonstrable benefit from standard clinical management, with reductions in adverse pregnancy outcomes in women with more severe hyperglycaemia and improvements in maternal infant metabolic outcomes in women with ‘milder’ GDM confirmed by high‐quality randomized controlled trial data.^1^

The COVID‐19 pandemic has prompted a review of procedures for the screening and diagnosis of hyperglycaemia during pregnancy. Stacey *et al*.^9^ have demonstrated no increase in stillbirth for women who are appropriately screened by OGTT and treated, but screening procedures are variably implemented and many high‐risk women are not screened. The HAPO follow‐up study highlights the longer‐term impact of antenatal hyperglycaemia on the risks of overweight, obesity and diabetes in both mother and child.^30^ Women who are appropriately screened and managed for GDM can now have pregnancy outcomes comparable to the background maternity population, but we lack contemporary data on their progression to type 2 diabetes and/or the longer‐term cardio‐metabolic outcomes. After the pandemic, larger population‐based studies should seek to evaluate whether the compliance benefits of having opportunistic tests such as HbA_1c_ and random plasma glucose performed in routine antenatal care settings can reduce the risks of perinatal death, especially among women in socially disadvantaged and higher‐risk ethnic groups. Various diagnostic thresholds at booking and at 28 weeks should be examined (separately and in combination) in relation to pregnancy outcomes alongside longer‐term cardio‐metabolic follow‐up programmes for women with treated GDM and their offspring.

In conclusion, the proposed changes to testing for hyperglycaemia during pregnancy facilitate identification of women at highest risk during the COVID‐19 pandemic peak, but should not be adopted long‐term. Future work should aim to identify pragmatic, evidence‐based alternatives to the OGTT, document the risks and benefits of opportunistic glycaemic testing in women from marginalized patient populations, and address longer‐term maternal and childhood cardio‐metabolic health outcomes.

## COMPETING INTERESTS

C.L.M., E.M.S., C.E.A., J.M., R.M.R., D.S., J.M.Y. and D.M. have no relevant conflicts of interest to declare. R.S.L. has received advisory boards/speaker fees for Novo Nordisk, Eli Lilly and Servier. H.R.M. has received honoraria for speaking engagements from Medtronic, Roche, Novo Nordisk, Eli‐Lilly and is a member of the Medtronic European Advisory Board.
