# Supplementary material for: Approaches to screening for hyperglycaemia in pregnant women during and after the COVID‐19 pandemic
Source: Diabet Med. 2020 Sep 21;38(1):e14380. doi: 10.1111/dme.14380 (PMC7436759; doi:10.1111/dme.14380)
Supplement: Supplementary file 1 [file DME-38-0-s001.docx]

**Table S1: The ability of a single RPG or FPG test (at 12w and 28w respectively) to predict the diagnosis of more severe forms of hyperglycaemia in pregnancy - diabetes in pregnancy and overt diabetes.**

The diagnosis of diabetes in pregnancy and overt diabetes are based upon the following criteria at any timepoint in pregnancy:

- Diagnosis of diabetes in pregnancy (WHO criteria, see below): Fasting glucose >7.0 mmol/l; 2-hour OGTT glucose >11.1 mmol/l; or random plasma glucose >11.1 mmol/l.
- Diagnosis of overt diabetes[2]: Fasting glucose >7.0 mmol/l; random plasma glucose >11.1 mmol/l;

Only women who had an OGTT and a RPG were included (n=3848) and no women had a random plasma glucose >11.1 mmol/l. The highest value in 17,736 women was 11.0 mmol/l (2004-2008).

| RANDOM PLASMA GLUCOSE at 12 weeks to predict more severe hyperglycaemia – OLD CUHFT DATA; n=17736 | | | | | | | | | |
| --- | --- | --- | --- | --- | --- | --- | --- | --- | --- |
|  | Diabetes in pregnancy (WHO) n=61  AUROC 0.61 (95% CI 0.53-0.70) | | | | | Overt diabetes (IADPSG) n=19  AUROC 0.56 (95% CI 0.43-0.69) | | | |
| Cutpoint | Sensitivity | Specificity | n | % +ve | | Sensitivity | Specificity | n | % +ve |
| > 7.0 mol/l | 68% | 41% | 1712 | 44% | | 63% | 41% | 1712 | 44% |
| > 7.5 mol/l | 66% | 45% | 1596 | 41% | | 56% | 45% | 1596 | 41% |
| > 7.8 mol/l | 60% | 48% | 1508 | 39% | | 44% | 48% | 1508 | 39% |
| > 8.0 mol/l | 57% | 54% | 1338 | 35% | | 44% | 54% | 1338 | 35% |
| > 8.5 mol/l | 43% | 72% | 831 | 22% | | 38% | 71% | 831 | 22% |
| > 9.0 mol/l | 32% | 83% | 515 | 13% | | 19% | 82% | 515 | 13% |
| > 9.5 mol/l | 26% | 90% | 311 | 8.1% | | 13% | 89% | 311 | 8.1% |
| > 10.0 mol/l | 15% | 95% | 158 | 4.1% | | 0.0% | 95% | 158 | 4.1% |
| FASTING PLASMA GLUCOSE at 28 weeks to predict more severe hyperglycaemia – OLD CUHFT DATA; n=17736 | | | | | | | | | |
|  | Diabetes in pregnancy (WHO) n=61  AUROC 0.90 (95% CI 0.86-0.95) | | | | Overt diabetes (IADPSG) n=19  AUROC 1.00 (95% CI 1.00-1.00)* | | | | |
| Cutpoint | Sensitivity | Specificity | n | % +ve | Sensitivity | | Specificity | n | % +ve |
| > 4.5 mol/l | 95% | 55% | 1758 | 46% | 100% | | 45% | 1758 | 46% |
| > 5.0 mol/l | 82% | 85% | 597 | 16% | 100% | | 81% | 597 | 16% |
| > 5.1 mol/l | 77% | 89% | 473 | 12% | 100% | | 85% | 473 | 12% |
| > 5.2 mol/l | 70% | 91% | 370 | 9.6% | 100% | | 91% | 370 | 9.6% |
| > 5.3 mol/l | 70% | 93% | 299 | 7.8% | 100% | | 93% | 299 | 7.8% |
| > 5.4 mol/l | 66% | 95% | 229 | 6.0% | 100% | | 94% | 229 | 6.0% |
| > 5.5 mol/l | 62% | 96% | 182 | 4.7% | 100% | | 96% | 182 | 4.7% |
| > 5.6 mol/l | 59% | 97% | 148 | 3.9% | 100% | | 97% | 148 | 3.9% |
| > 6.0 mol/l | 49% | 99% | 78 | 2.0% | 100% | | 98% | 78 | 2.0% |

World Health Organisation (2013) Diagnostic Criteria and Classification of Hyperglycaemia First Detected in Pregnancy.
